# Supplementary material for: Whole-Genome Identification and Analysis of Multiple Gene Families Reveal Candidate Genes for Theasaponin Biosynthesis in Camellia oleifera
Source: Int J Mol Sci. 2022 Jun 7;23(12):6393. doi: 10.3390/ijms23126393 (PMC9223445; doi:10.3390/ijms23126393)
Supplement: Supplementary file 1 [file ijms-23-06393-s001.zip › ijms-1759733-supplementary/Supplementary Figures and tables/Supplementary Figures.pdf]

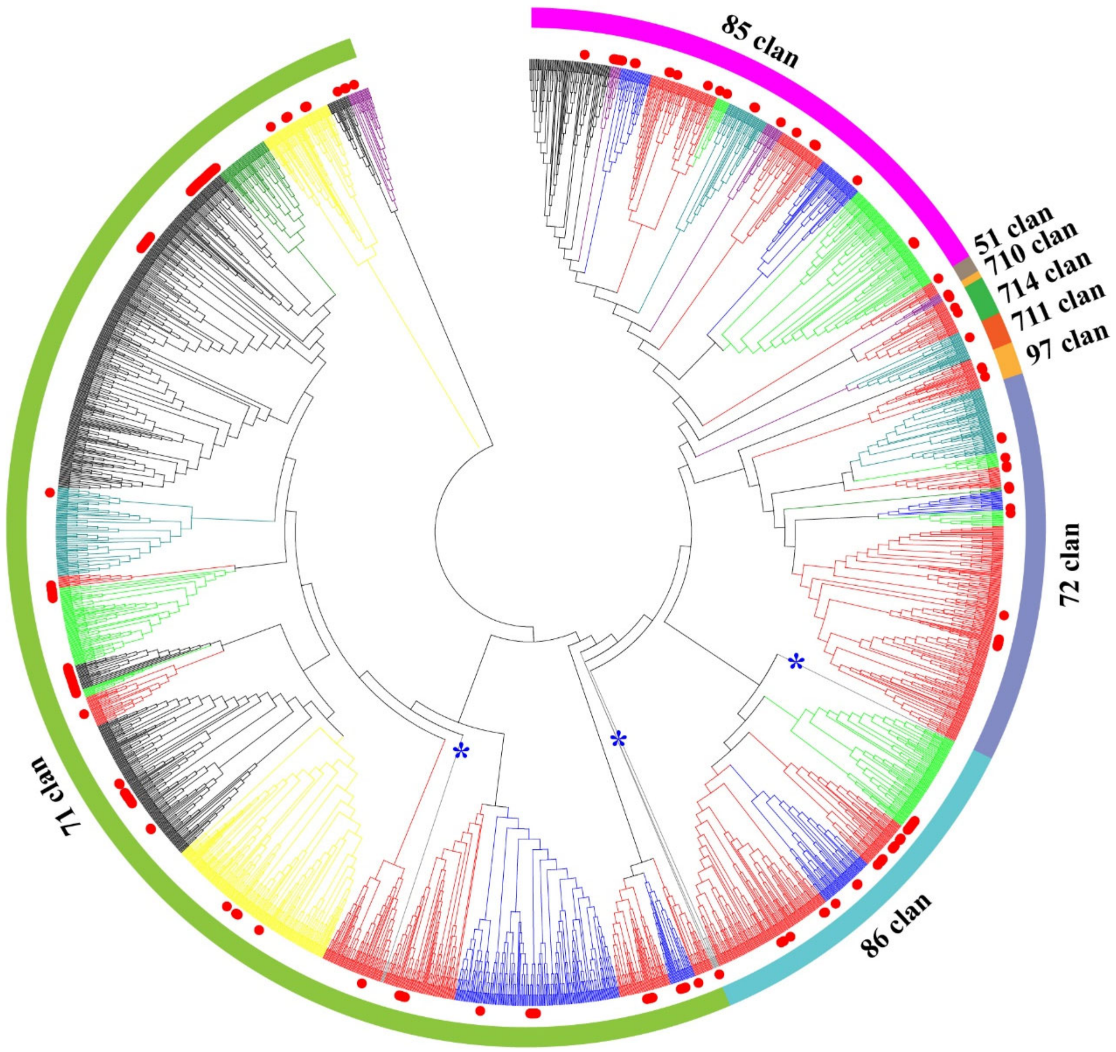

Figure S1. Phylogenetic tree of CYP450s from Camellia oleifera, Arabidopsis thaliana, Camellia sinensis, Actinidia chinensis, Panax ginseng, and Medicago truncatula. Different families and clans were divided by different colors. The representative CYP450 family members from Arabidopsis and P. ginseng are marked with red dots. Blue asterisk (*) indicates the proteins that do not fall into any of the families described above.

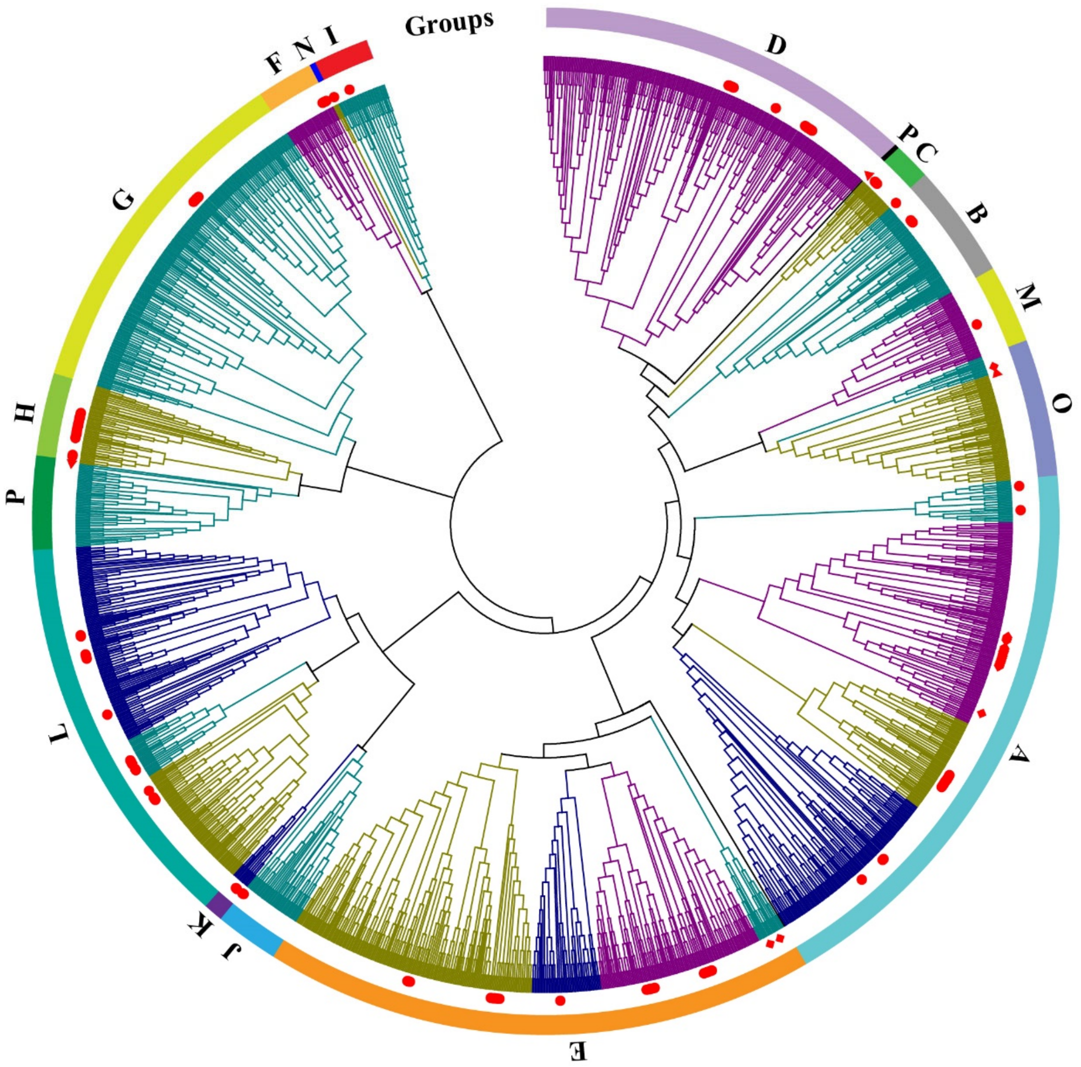

Figure S2. Phylogenetic tree of UGTs from Camellia oleifera, Arabidopsis thaliana, Camellia sinensis, Actinidia chinensis, Panax ginseng, and Medicago truncatula. Different families and groups are divided by different colors. The representative UGT family members from Arabidopsis, P. ginseng, and maize are marked with red dots, diamonds, and triangles, respectively. The accession numbers of three representative UGTs from maize are GRMZM2G042865 (Q group), GRMZM2G120016 (O group), and GRMZM5G834303 (P group).

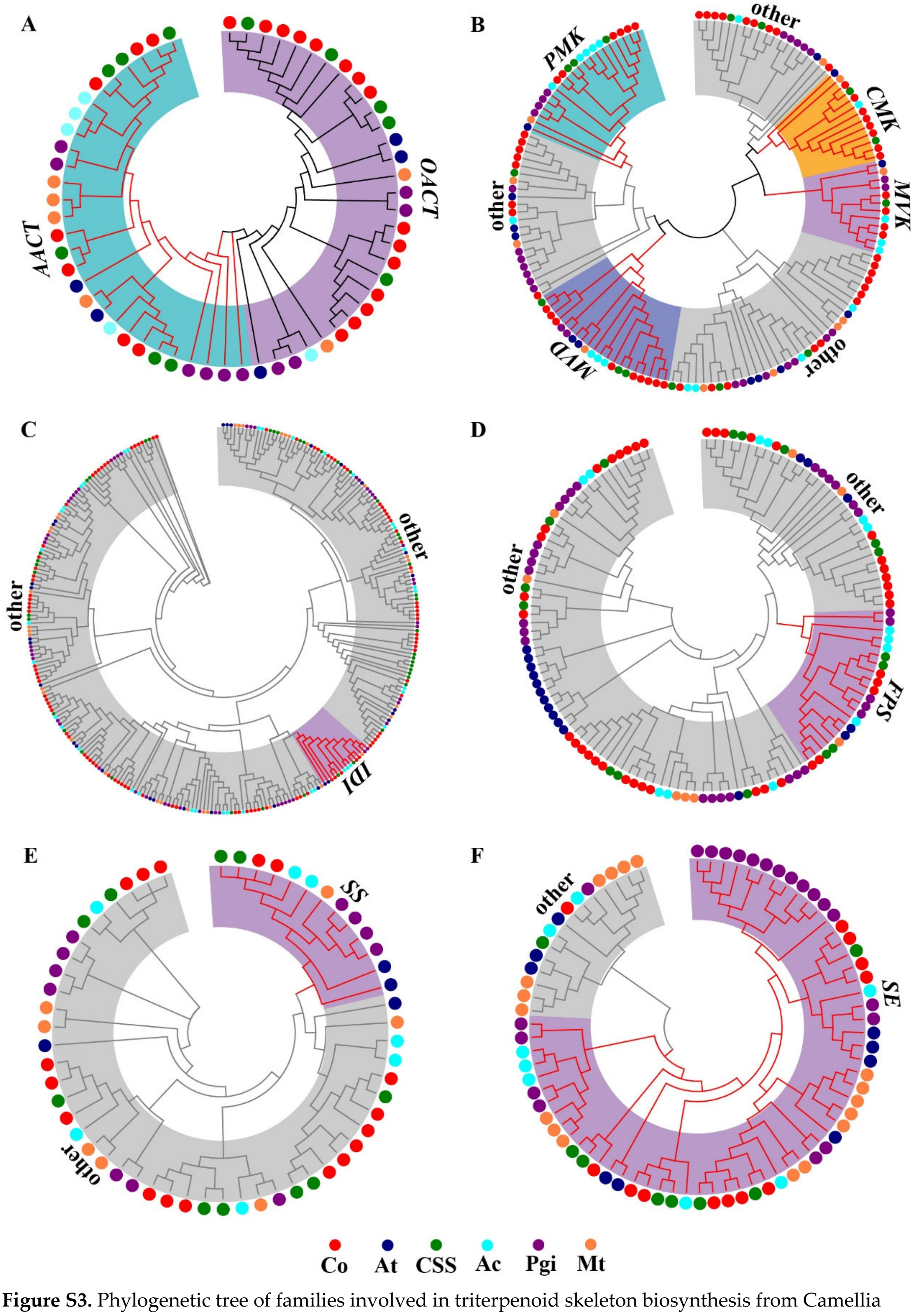

Figure S3. Phylogenetic tree of families involved in triterpenoid skeleton biosynthesis from Camellia oleifera (Co), Arabidopsis thaliana (At), Camellia sinensis (CSS), Actinidia chinensis (Ac), Panax ginseng (Pgi), and Medicago truncatula (Mt). Different families are divided by different colors, and 'others' represents other proteins possessing the same domains as our target families.
